# Dietary Inclusion of Stabilized Partially Destoned Olive Cake in Grazing Dairy Ewes Preserves Cheese Quality While Modulating the Sensory Profile of Pecorino Siciliano PDO Cheese

**DOI:** 10.3390/foods15183186

**Published:** 2026-09-09

**Authors:** Mahmood Ul Hassan, Riccardo Gannuscio, Giuseppe Maniaci, Gabriele Busetta, Marco Alabiso, Elena Franciosi, Luca Settanni, Raimondo Gaglio, Massimo Todaro

**Affiliations:** 1 Department of Agricultural, Food and Forestry Science (SAAF), Università di Palermo, Viale delle Scienze 13, 90128 Palermo, Italy; mahmoodul.hassan@unipa.it (M.U.H.); riccardo.gannuscio@unipa.it (R.G.); gabriele.busetta@unipa.it (G.B.); marco.alabiso@unipa.it (M.A.); luca.settanni@unipa.it (L.S.); raimondo.gaglio@unipa.it (R.G.); massimo.todaro@unipa.it (M.T.); 2 Research and Innovation Centre, Fondazione Edmund Mach (FEM), Via E. Mach 1, 38098 San Michele All’Adige, Italy; elena.franciosi@fmach.it

**Keywords:** olive cake by-product, dairy ewes, cheese, sensory traits, fatty acids, microbiology

## Abstract

This study evaluated the effects of dietary inclusion of stabilized, partially destoned olive cake (PDOC) on bulk milk characteristics and the physicochemical, microbiological, and sensory properties of Pecorino Siciliano PDO and Ricotta cheeses. A total of 124 Valle del Belice dairy ewes were randomly assigned to two homogeneous groups according to parity (≥3rd lambing), days in milk (60 ± 5), and milk yield (1.48 ± 0.4 kg/day). Both groups grazed the same pasture and were supplemented with 0.5 kg/head/day of concentrate, either without PDOC (CTR) or containing 17% PDOC on an as-fed basis (EXP). The concentrates were formulated to be isoenergetic. Milk was collected fortnightly from morning and evening milkings, pooled by treatment, and used for cheesemaking. Bulk milk, feeds, and PDOC were analyzed for chemical composition. Fresh Ricotta and 6-month-ripened Pecorino Siciliano PDO cheeses were evaluated for composition, fatty acid (FA) profile, microbiological characteristics, and sensory properties. Data were analyzed using the MIXED model. Dietary treatment did not affect bulk milk composition but improved milk coagulation properties. Likewise, composition, titratable acidity, color, and hardness were unaffected by dietary treatment. Total phenolic content (TPC), peroxide value, and thiobarbituric acid-reactive substances in Pecorino cheese did not differ between treatments, whereas Ricotta from the CTR group unexpectedly exhibited a higher TPC than that from the EXP group (*p* < 0.01). Dietary inclusion of PDOC affected only a limited number of fatty acids, resulting in lower (*p* < 0.05) total MUFA and MCFA contents in the EXP group. Raw milk from EXP ewes showed higher counts of total mesophilic bacteria and lactic acid bacteria (*p* < 0.05); however, microbial populations became comparable after contact with the wooden vat and remained similar throughout ripening (*p* > 0.05), with no pathogenic bacteria detected in either group. Sensory analysis revealed differences in the sensory profile of Pecorino cheeses produced from EXP milk (*p* < 0.05), although overall acceptability did not differ between treatments. In conclusion, dietary inclusion of PDOC in dairy ewe diets appears to preserve the composition, safety, and overall acceptability of Pecorino Siciliano PDO and Ricotta cheeses.

## 1. Introduction

Current global challenges, including climate change, natural resource depletion, and increasing competition in international markets, necessitate a transition from a linear production model based on production, consumption, and disposal to a circular system that emphasizes resource reuse and recycling, thereby closing material loops [1]. In this context, the valorization of agro-industrial by-products represents a key strategy for promoting circular economy models, reducing competition for land and water resources, and supporting more sustainable livestock production [2]. The Mediterranean region accounts for approximately 95% of the world’s olive (*Olea europaea* L.) cultivation and contributes nearly 70% of global olive oil production [3], with Italy representing about 15% of total European output. However, olive processing generates substantial quantities of by-products; for every 1000 kg of olives processed, approximately 800 kg of waste is produced [4]. These residues are often underutilized or improperly managed, potentially posing significant environmental concerns [5].

Among olive-processing by-products, partially destoned olive cake (PDOC) has emerged as a promising feed ingredient for ruminants. Partial removal of the stones reduces lignin content and improves digestibility compared with conventional olive cake [6,7,8]. Olive cake is also rich in fiber, oleic acid, and polyphenolic compounds and contains moderate levels of crude protein [9,10], making it a potentially suitable alternative to conventional feed resources in dairy sheep systems.

From a human nutrition perspective, reducing dietary saturated fatty acid (SFA) intake has long been recommended to improve cardiovascular health. Dairy products are important contributors to SFA intake and have traditionally been associated with increased plasma cholesterol levels [11]. Accordingly, dietary guidelines from the USDA recommend limiting saturated fat consumption, including that derived from dairy products [12]. Consequently, strategies aimed at modifying the fatty acid (FA) profile of milk and dairy products have attracted increasing interest.

Dietary manipulation of dairy animals can modify milk fat composition, potentially reducing medium-chain saturated fatty acids (MCSFA), which have traditionally been considered hypercholesterolemic, while increasing fatty acids with recognized nutritional interest, including n-3 polyunsaturated fatty acids (PUFA) and conjugated linoleic acid (CLA), particularly cis-9 and trans-11 C18:2 [13]. Dietary supplementation with unsaturated plant sources has also been shown to modify the FA profile of ruminant milk [14]. These effects are particularly relevant for dairy products because changes in milk fat composition can be transferred, at least partially, to derived cheeses and may influence their nutritional characteristics.

Olive-processing by-products are of particular interest in this context because their high oleic acid content and other bioactive compounds may contribute to changes in the FA composition of milk and dairy products. Previous studies have reported that the inclusion of olive cake or related olive by-products in ruminant diets can decrease short- and medium-chain saturated fatty acids while increasing unsaturated fatty acids, including oleic acid, vaccenic acid, and CLA [15,16]. In cheese, these changes have been associated with improved nutritional indices, including higher UFA/SFA ratios, increased n-3 and n-6 FA contents, and reduced atherogenic and thrombogenic indices, together with modifications in sensory properties during ripening [15].

Although the effects of olive by-products on milk and dairy products have been investigated in several ruminant production systems, their effects on traditional and seasonal dairy products obtained from grazing dairy ewes remain less well documented. In particular, previous research has generally focused on milk or cheeses with relatively short ripening periods, whereas information on the effects of olive by-product supplementation on long-ripened traditional ewe cheeses is more limited [15,17,18].

This aspect is particularly relevant for Pecorino Siciliano PDO, a traditional cheese whose characteristics are influenced not only by milk composition but also by cheesemaking procedures and the extended ripening process. The six-month ripening period adopted for Pecorino Siciliano PDO in the present study provides an opportunity to evaluate whether potential dietary effects on milk composition and fatty acid profile are retained, attenuated, or otherwise expressed in the final ripened cheese. Moreover, the production of Ricotta from the whey generated during cheesemaking allows the evaluation of the potential effects of the dietary treatment on an additional traditional dairy product.

Given that olive-processing by-products, in combination with grazing-based feeding systems, may modify the milk FA profile by increasing the proportion of long-chain fatty acids, particularly oleic and linoleic acids, these dietary changes could potentially influence lipid-related processes occurring during cheese ripening. Such effects may, in turn, contribute to differences in the sensory characteristics of the final product.

A further objective was to assess whether the inclusion of stabilized PDOC could be used as a sustainable feeding strategy without negatively affecting the technological, microbiological, nutritional, or sensory quality of traditional dairy products. We hypothesized that dietary supplementation with stabilized partially destoned olive cake would produce ripened cheeses with a distinctive sensory profile, potentially characterized by enhanced aromatic complexity and modified flavour intensity.

## 2. Materials and Methods

The study was carried out in compliance with Italian Legislative Decree No. 26/2014, which implements Directive 2010/63/EU concerning animal welfare and good clinical practice. The experimental protocol was approved by the Animal Welfare Body (OPBA) of the University of Palermo (protocol number: UNPA-CLE-Prot.117.247, 12 July 2024).

### 2.1. Animals, Experimental Design, and Diets

The feeding trial lasted 14 weeks, comprising an initial 2-week adaptation phase and a subsequent 12-week period during which samples were collected. The study was performed on lactating Valle del Belice dairy ewes kept on a farm in Castronovo di Sicilia, Italy, located within the traditional production area of Pecorino Siciliano PDO cheese. A total of 124 ewes were included in the experiment and assigned to two groups of 62 animals each according to dietary treatment (CTR and EXP). The groups were balanced for parity (≥3rd lambing), days in milk (60 ± 5 d), and daily milk yield (1.48 ± 0.4 kg/d).

Both groups grazed on the same pasture and were provided with isoenergetic concentrates differing in the inclusion of partially destoned olive cake. The control group (CTR) received a concentrate without partially destoned olive cake, whereas the experimental group (EXP) was supplied with a concentrate containing 17% stabilized partially destoned olive cake on an as-fed basis. The ingredients used in the formulation of the two concentrates are listed in Table 1, and their chemical composition is reported in Table 2.

The concentrates were administered once a day at 16:00 h at a rate of 0.5 kg/ewe on an as-fed basis. All the animals completely consumed the amount provided. Fresh water was continuously available ad libitum. Voluntary feed intake (VFI) during grazing was not directly measured; however, based on the data reported in the literature on the VFI of green forage by Valle del Belice ewes [19,20], it is possible to estimate a forage-to-concentrate ratio of 75:25. Animal health was monitored throughout the trial, and no relevant health problems were observed.

### 2.2. Partially Destoned Olive Cake Production Process

Partially destoned olive cake (PDOC) used in the study was provided by Azienda Olearia Consoli Pasquale & F.lli s.n.c., located in Catania (Sicily, southern Italy). Fresh olives were processed using a 2-phase extraction method to produce olive oil, yielding wet olive cake with approximately 80% moisture. The wet olive cake (OC) was stabilized with hydrochloric acid (HCl; E507) and stored in containers for 180 days. Subsequently, a centrifugation step (2-phase extraction) separated the liquid and solid fractions, resulting in OC with ~55% moisture. The OC was then exposed to sunlight for 5 days, reducing its moisture content to approximately 8–10%. Afterward, it was ground and passed through a cyclone system to achieve partial destoning [7].

A limitation of the present study is that detailed information on the HCl treatment applied during the processing of the PDOC was not available. The specific conditions and characteristics of this treatment are considered proprietary information by the manufacturer and therefore could not be disclosed. Consequently, although the PDOC used in this study has been previously evaluated for its suitability as a feed ingredient, the lack of detailed information on the processing treatment limits the possibility of fully characterizing the technological properties of the product and their potential influence on its nutritional and biological effects.

### 2.3. Dairy Products Manufacturing

Every two weeks, milk from the CTR and EXP groups was processed separately for cheesemaking. For each group, the bulk milk obtained from the morning and evening milkings was collected and used for the production of Pecorino Siciliano PDO and Ricotta. Cheesemaking was carried out at a private dairy facility in Castronovo di Sicilia (Palermo, Italy), affiliated with the Consortium for the Valorization and Promotion of Pecorino Siciliano PDO cheese. Bulk raw ewe milk was processed every two weeks (6 batches in total) into Pecorino Siciliano PDO and Ricotta cheeses using traditional technology, as described by Todaro et al. [21] and Mangione et al. [22] for Pecorino Siciliano and Ricotta cheese, respectively. Briefly, 100 L of bulk milk was brought to 38 °C and transferred to a 12-year-old chestnut wood vat. The milk was gently agitated by hand for 5 min before lamb rennet paste was added (20 g; Rennet Regional Consortium, Poggioreale, Italy). Coagulation was allowed to proceed for 40 min. Subsequently, 10 L of water heated to 74 °C was added to the intact coagulum to promote syneresis. The coagulum was then broken with a wooden paddle locally known as a “rotula”, producing grains approximately 3–7 mm in diameter, comparable in size to rice grains. Once the whey had been drained, the curd was manually pressed into 10-kg rattan baskets. For each cheesemaking batch, one cheese from the CTR group and one cheese from the EXP group were cooked for 3 h at 70 °C in hot scotta whey obtained as a by-product of Ricotta manufacture. After cooking, the cheeses were kept for 24 h before being salted in saturated brine for a further 24 h. Ripening was subsequently carried out for 6 months in a storage chamber maintained at 16 °C and 85% relative humidity (RH). For Ricotta production, after curd separation, the resulting whey was filtered, transferred into a large kettle, and heated to 45 °C, after which salt (0.5%) was added. Subsequently, raw sheep milk (8%) was added at approximately 50 °C. The mixture was then heated to approximately 85 °C until the flocculated proteins rose to the surface. Once they had fully risen to the surface, the Ricotta was manually collected and placed into perforated plastic cylindrical containers (baskets called “fuscelle”) to allow the scotta whey to drain. The Ricotta was left to drain at room temperature and sampled 30 min after production. The cheese-making process was repeated six times (two productions per month) during April, May, and June, when green forage availability is highest, representing the period traditionally considered most suitable for producing high-quality Pecorino Siciliano PDO cheese.

### 2.4. Feed Sampling and Chemical Analysis

Pasture and concentrate (CTR and EXP) samples were collected every two weeks and transferred to the Animal Nutrition Laboratory of the Department of SAAF at the University of Palermo. The pasture samples were then freeze-dried. Freeze-dried pasture and concentrate samples were ground in a Willey mill (Thomas Scientific, Swedesboro, NJ, USA) using a 1.0-mm sieve [23] for analyses. All feeds were analyzed for dry matter (DM method, 967.03), ash (method, 942.05), ether extract (EE method, 920.29), and crude protein (CP, TN × 6.25, method 988.05), following AOAC [23] methods. The fiber fractions NDFom (neutral detergent fiber exclusive of residual ash), ADFom (acid detergent fiber exclusive of residual ash), and ADL (acid detergent lignin) were analyzed following the procedure described by Van Soest et al. [24]. Non-fiber carbohydrates (NFCs) were calculated as NFC = 100 − [CP + EE + Ash + NDFom] as described by Maniaci et al. [25]. The total phenolic content (TPC) was determined using the optimized Folin–Ciocâlteu method [26], with slight modifications [27]. Fatty acids composition of PDOC, pasture, and concentrates (CTR and EXP) were determined as described below.

### 2.5. Bulk Milk and Dairy Products: Physicochemical Analyses

Fresh bulk milk samples were analyzed for lactose, fat, protein, casein, urea, and somatic cell count (SCC) using the infrared method (Combi-foss 6000, Foss Electric, Hillerød, Denmark); total bacterial count (TBC) was measured using a BactoScan instrument (Foss Electric); and pH using an HI 9025 pH meter (Hanna Instruments, Ann Arbor, MI, USA). Total nitrogen (TN), non-casein nitrogen (NCN), and non-protein nitrogen (NPN) fractions were determined according to International Dairy Federation (IDF) standard procedures [28,29]. Total protein was calculated as TN × 6.38. Casein was calculated as [TN − (NCN × 0.994)] × 6.38, and whey protein was calculated as [NCN − NPN] × 6.38. Milk coagulation properties were determined using a Formagraph (Foss Electric, Hillerød, Denmark). Milk samples (10 mL) were analyzed at 35 °C after addition of 0.2 mL of diluted rennet solution (1.6:100 *v*/*v*; rennet strength 1:15,000; Chr. Hansen, Parma, Italy). The parameters recorded were rennet coagulation time (r, min), curd-firming time (k20, min), and curd firmness at 30 min (a30, mm).

Cheese samples were collected after 6 months of ripening for physical–chemical analyses. Titratable acidity of the cheeses was determined by the Soxhlet–Henkel method, expressed as °SH/50 mL. Two subsamples were taken from the inner portion of each cheese using a sterile knife. Surface color was measured in duplicate using a Minolta Chroma Meter CR-300 (Minolta, Osaka, Japan) with illuminant C, and results were expressed in CIE Lab* coordinates: lightness (L*), redness (a*), and yellowness (b*) [30]. Cheese texture was evaluated using an Instron 5564 universal testing machine (Instron, Trezzano sul Naviglio, Italy) by measuring maximum compressive stress (N/mm^2^) on 2 × 2 × 2 cm samples equilibrated at 22 °C.

Freeze-dried cheese and ricotta samples were analyzed for dry matter, fat, crude protein (TN × 6.38), and ash according to AOAC methods [21].

### 2.6. Total Phenolic Content and Lipid Oxidation of Dairy Products

A methanolic extraction of phenolic was prepared according to the method of Rashidinejad et al. [31] with slight modifications, while the TPC was measured using the Folin-Ciocâlteu method [32,33].

The primary lipid oxidation of dairy fat was evaluated by determining the peroxide value (POV) (g gallic acid equivalent (GAE)/kg DM) [34], while secondary lipid oxidation was evaluated by determining thiobarbituric acid-reactive substances (TBARs) [35].

### 2.7. Fatty Acid Composition of Feed and Dairy Products

Fatty acids in lyophilized pasture, concentrate, and Pecorino Siciliano and Ricotta cheese samples (100 mg) were directly methylated using a bimethylation procedure as described by Lee and Tweed [36]. Samples were incubated in 1 mL hexane and 2 mL of 0.5 M sodium methoxide at 50 °C for 15 min, followed by addition of 1 mL of 5% HCl in methanol and incubation at 50 °C for 15 min. Fatty acid methyl esters (FAMEs) were extracted with 1.5 mL hexane. FAMEs were analyzed using an HP 6890 gas chromatograph (Agilent Technologies, Santa Clara, CA, USA) equipped with a flame-ionization detector. One microliter of sample was injected using an autosampler. Separation was performed on a CP-Sil 88 capillary column (100 m × 0.25 mm i.d., 0.25 µm film thickness; Chrompack, Middelburg, the Netherlands). Injector and detector temperatures were set at 255 °C and 250 °C, respectively. Helium was used as the carrier gas at a constant flow rate of 0.7 mL/min (linear velocity 14 cm/s; inlet pressure 158.6 kPa). Hydrogen and air flows to the detector were 40 and 400 mL/min, respectively. The oven temperature program was 70 °C for 1 min, increased to 100 °C at 5 °C/min (held 2 min), then to 175 °C at 10 °C/min (held 40 min), and finally to 225 °C at 5 °C/min (held 45 min). Fatty acids were identified by comparison of retention times with a commercial FAME standard mixture (Nu-Chek-Prep, Elysian, MN, USA). Conjugated linoleic acid isomers were identified using standards of C18:2 c9t11 (rumenic acid) and C18:2 c10t12 methyl esters (Sigma-Aldrich, Milan, Italy), as well as published isomeric profiles [37,38]. Total fatty acid content was quantified using C23:0 (Sigma-Aldrich) as an internal standard (4 mg/g lyophilized cheese).

### 2.8. Microbiological Analysis

All samples collected during the production of Pecorino Siciliano PDO cheese, from wooden vat biofilms to the cheese after six months of ripening, were subjected to a thorough microbiological analysis. One mL of liquid samples (wooden vat biofilms, milk and milk after contact) was directly serially decimally diluted in Ringer’s solution (Sigma-Aldrich, Milan, Italy), while 15 g of solid samples (curd, fresh cheese and cheese after six months of ripening) was transferred into sterile bags (BagFilter P, Interscience, Saint Nom, France) and homogenized with 135 mL of sodium citrate (2% *w*/*v*) solution using a stomacher (BagMixer^®^ 400, Interscience) at the highest speed for 2 min. The resulting homogenates were subsequently diluted in tenfold steps prior to plating. From each sample, suitable dilutions were inoculated onto selective agar media to enumerate and characterize specific microbial groups: total mesophilic microorganisms (TMMs) were inoculated on Skim Milk Agar (SMA) incubated at 30 °C for 72 h; thermophilic and mesophilic coccus LAB were poured in Medium 17 (M17) agar containing 5 g/L of lactose and incubated for 48 h at 44 °C and 30 °C, respectively; and thermophilic and mesophilic LAB rods were inoculated on de Man-Rogosa-Sharpe agar acidified to pH 5.4 with 5 M lactic acid and incubated for 48 h at 44 °C and 30 °C. All samples were also examined for major pathogenic bacteria: *Escherichia coli* and coagulase-positive staphylococci (CPS) were enumerated following the ISO 7251 [39] and ISO 6888-2 [40], while *Listeria monocytogenes* and *Salmonella* spp. were enumerated following the ISO 11290-2, 2017 and ISO 6579-2, 2017. In addition, the detection of *L. monocytogenes* and *Salmonella* spp. was performed according to ISO 11290-1 [41] and ISO 6579-1 [42] guidelines, respectively. The lower limit of quantification for the enumeration procedures was <1 CFU/mL in liquid samples and <2 log CFU/g in solid matrices. Conversely, qualitative detection methods were able to identify the target microorganisms at levels corresponding to 0 log CFU/mL or g in both sample types. Each analysis was performed in duplicate using media and supplements purchased from Lickson (Vicari, Italy).

### 2.9. Culture-Independent Analysis of Total Bacterial Community

Microbial DNA was extracted from fresh cheese and Pecorino Siciliano PDO cheese samples using the DNeasy PowerFood Microbial Kit (QIAGEN, Hilden, Germany), following the standard protocol. For each trial, DNA from replicate samples was pooled to form composite samples. DNA concentrations were determined using the NanoDrop ND 1000 spectrophotometer (NanoDrop Technologies, Wilmington, DE, USA). DNA integrity and concentration were evaluated via agarose gel electrophoresis and UV/Vis spectrophotometry. After DNA extraction, the V3–V4 hypervariable regions of the bacterial 16S rRNA gene were amplified using primers 341F (5′-CCTACGGGNGGCWGCAG-3′) and 806R (5′-GACTACNVGGGTWTCTAATCC-3′) and sequenced on the Illumina MiSeq system at the Fondazione Edmund Mach (FEM, San Michele a/Adige, Italy) sequencing platform, yielding 250 bp paired-end reads. After sequencing, raw reads were analyzed using the Quantitative Insights Into Microbial Ecology (Qiime2, version 2018.2) [43] software. Quality and chimera filtering were performed using the qiime DADA2 denoise-paired script [44]. Representative bacterial sequences were aligned using MAFFT and used for phylogenetic reconstruction in FastTree, leveraging the alignment and phylogeny plugins [45]. Taxonomic and compositional analyses for bacteria were conducted using the feature-classifier plugin (available at svg with https://github.com/qiime2/q2-feature-classifier, accessed on 30 June 2026). A pre-trained Naive Bayes classifier, based on the Greengenes 13_8 99% Operational Taxonomic Units (OTUs) database which had been previously trimmed to the V4 region of 16S rDNA, bound by the 341F/805R primer pair, was applied to paired-end sequence reads to generate taxonomy tables. The MiSeq Illumina sequencing data have been deposited in the NCBI Sequence Read Archive (SRA) and are accessible under Accession Number PRJNA1494385.

### 2.10. Sensory Analysis

The sensory profile of the CTR and EXP cheeses after six months of ripening was assessed by a trained panel comprising 12 assessors (six males and six females, aged 21–65 years), all with previous experience in cheese sensory evaluation. Sensory testing was performed in individual booths, and the assessors received specific training in accordance with ISO 4120 [46]. Before evaluation, the cheese samples were allowed to equilibrate to approximately 20 °C for 1 h and were subsequently cut into 3 × 3 × 3 cm cubes. Each sample was assigned a random code and presented to the assessors in a randomized sequence. Ten descriptive attributes were evaluated, covering four sensory dimensions: appearance, aroma, taste, and texture. Attribute intensity was rated on a 9-cm line scale ranging from 1 to 9, as described by Todaro et al. [21].

### 2.11. Statistical Analysis

Bulk milk, Ricotta, and cheese composition and fatty acid profiles were analyzed using a mixed model, considering diet (D) as a fixed factor and date of cheese-making as a random factor, using the MIXED procedure of SAS 9.2 software [47]. The experimental unit was the date of cheese-making, with six independent replicates per dietary treatment. The statistical model was:

Y_ijk_ = μ+ D_i_ +Date_ij_ + ε_ijk_

where Y_ijk_ is the observed value, µ is the overall mean, D_i_ is the fixed effect of diet (i = CTR, EXP), Date_ij_ is the random effect of date of cheese-making (j = 1, ..., 6) and ε_ijk_ is the random residual error. Differences between means were assessed using Tukey’s post hoc test. Statistical significance was declared at *p* < 0.05.

Sensory data were analyzed using a linear mixed-effects model, with diet included as a fixed effect and panelist as a random effect.

Y_ijk_ = μ + D_i_ + P_ij_ + ε_ijk_

where Y_ijk_ are observations, µ is the overall mean, D_i_ is the fixed effect of diet (i = CTR, EXP), P_ij_ is the random effect of assessors (j = 1–12), and ε_ijk_ is the random residual error. Differences between means were assessed using Tukey’s post hoc test. Statistical significance was declared at *p* < 0.05.

## 3. Results and Discussion

### 3.1. Chemical Composition of Feeds

The chemical composition and fatty acid profile of the concentrates, partially destoned olive cake (PDOC), and pasture are reported in Table 2. The inclusion of PDOC in the experimental (EXP) concentrate (17%, as-fed basis) modified its chemical composition compared with the control (CTR) concentrate. Although the two concentrates were designed to have a comparable estimated energy content (approximately 1800 kcal/kg DM), the EXP diet was characterized by greater amounts of crude protein (CP; 20.97 vs. 19.06% DM) and ether extract (EE; 5.68 vs. 2.75% DM) than the control concentrate. The greater EE content of the EXP concentrate reflected the relatively high lipid concentration of PDOC (21.70% DM). The EXP concentrate also showed higher fiber fractions, with greater concentrations of neutral detergent fiber (NDFom; 64.27 vs. 60.12% DM) and acid detergent lignin (ADL; 22.08 vs. 19.35% DM), likely reflecting the presence of lignified olive stone residues. Total polyphenol content was greater in the EXP concentrate than in the CTR concentrate, reaching 5.60 mg GAE/g DM compared with 3.79 mg GAE/g DM, respectively.

The chemical composition of PDOC, particularly its crude protein (CP) content, differed from that reported by Molina-Alcaide and Yáñez-Ruiz [4] and Terramoccia et al. [48], confirming the considerable compositional variability commonly observed among olive cake by-products. This variability has been attributed to differences in processing techniques, olive cultivar, environmental conditions, fruit maturity, and production year [49,50]. Pasture was characterized by a high proportion of polyunsaturated fatty acids (PUFA; 63.35% of total fatty acids), mainly α-linolenic acid (35.38% of total fatty acids), and by a relatively high total polyphenol content (14.34 mg GAE/g DM), consistent with previous reports [22,51].

Considering that two isoenergetic concentrates were formulated, the inclusion of PDOC also resulted in significant changes in other parameters, such as crude protein, ether extract, and fatty acid composition. Therefore, when discussing the effects of PDOC on the chemical, microbiological, and sensory characteristics of dairy products the potential combined effects of these changes should also be taken into account.

### 3.2. Physicochemical and Technological Characteristics of Bulk Milk

Dietary treatment did not affect bulk milk composition or technological parameters (Table 3). Somatic cell count (SCC) was higher in the EXP group (*p* < 0.05), although this parameter is known to be influenced by a wide range of nutritional and non-nutritional factors, including animal health status, management practices, environmental conditions, and stage of lactation. Therefore, the observed increase in SCC should not be attributed solely to the dietary treatment [52]. Milk composition is generally influenced by dietary factors [53]; however, the inclusion of PDOC in the EXP concentrate did not affect milk composition in the present study.

In contrast, PDOC supplementation appeared to improve the cheese-making aptitude of the milk, as indicated by the higher curd firmness (*p* < 0.01) and shorter curd-firming time (*p* < 0.05) observed in EXP bulk milk. These coagulation properties may contribute positively to the technological suitability of milk for the production of ripened cheese.

The present results are consistent with those of previous studies showing that milk composition was not adversely affected by the inclusion of dried olive cake at levels of 15–20% of dietary DM in dairy cow and ewe diets. Similarly, the inclusion of olive cake obtained from either two-phase (135 g/kg DM) or three-phase (112.5 g/kg DM) olive oil extraction processes in diets for Comisana dairy ewes did not adversely affect milk composition [15,54,55,56,57].

### 3.3. Physical Properties and Chemical Composition of Ricotta and Ripened Pecorino Cheese

The physical properties and chemical composition of Ricotta and ripened Pecorino cheeses are presented in Table 4.

Dietary inclusion of olive cake by-products did not affect the chemical composition of either ripened Pecorino or Ricotta cheese, in agreement with Abbeddou et al. [58], who reported no effect of olive cake supplementation on the composition of farmhouse-type fresh ewe cheese, and with previous studies showing that olive cake supplementation has little influence on milk composition [15,59]. Likewise, no significant differences among dietary treatments were observed in the titratable acidity, color parameters (L*, a*, and b*), or hardness of PDO Pecorino cheese. Furthermore, the composition of Ricotta was consistent with the limited information available for this traditional Sicilian dairy by-product in the literature [22].

Previous studies have reported that olive cake supplementation increased the total phenolic content (TPC) of cheese [59,60,61], an effect generally attributed to the transfer of dietary polyphenols from feed to milk and, subsequently, to cheese. In contrast, no differences in cheese TPC were detected between treatments in the present study. Likewise, peroxide value (POV) and thiobarbituric acid reactive substances (TBARS), two indicators of lipid oxidation, were unaffected by dietary treatment. Given the close relationship between phenolic antioxidants and oxidative stability, the absence of an increase in cheese TPC may explain the lack of differences in these oxidative stability markers. This finding suggests that its relatively low inclusion level in the total diet (forage-to-concentrate ratio of 75:25) may not have provided sufficient polyphenols to enhance the antioxidant status of the cheese. Moreover, results from the same experimental trial, previously reported by Hassan et al. [62], showed no significant effects of diet on ruminal pH, total volatile fatty acid (VFA) concentration, NH_3_ concentration, protozoal counts, or CH_4_ production, suggesting that the overall ruminal fermentation profile was not markedly affected despite the reduction in feed degradability. The limited impact of PDOC on ruminal fermentation may also have contributed to the lack of detectable changes in the transfer of bioactive compounds to milk and cheese. This differs from previous studies in dairy cows, in which supplementation with polyphenol-enriched olive cake increased the total polyphenol content (TPC) of cheese [60,61], likely reflecting differences in the phenolic composition of the dietary supplement.

In contrast, dietary treatment significantly affected the TPC of Ricotta cheese (*p* < 0.01), with higher values observed in the CTR group. This unexpected finding may be related to differences in the polyphenolic composition of whey, which could affect the transfer and retention of individual phenolic compounds during Ricotta manufacture [63]. Polyphenols are known to interact with milk proteins, although the extent of these interactions depends on several factors, including pH, ionic strength, and the structural characteristics of both proteins and phenolic compounds. Furthermore, the heat treatment applied during Ricotta manufacture may alter protein conformation and polyphenol solubility, thereby modifying protein–polyphenol interactions and, consequently, the retention of phenolic compounds in the final product [63].

### 3.4. Fatty Acid Composition of Ricotta and Ripened Pecorino Cheeses

Dietary inclusion of olive cake affected only a limited number of fatty acids, most of which were present in relatively low proportions in both Ricotta and 6-month-ripened Pecorino cheese (Table 5 and Table 6). In the 6-month-ripened Pecorino cheese, the EXP group showed significantly lower proportions of medium-chain unsaturated fatty acids, namely C10:1, C12:1, C14:1, and C16:1 (*p* < 0.05), which contributed to the significantly lower total MUFA and MCFA content observed in the EXP group (*p* < 0.05).

The reduction in individual unsaturated fatty acids was accompanied by a significant decrease in total MCFA in Pecorino cheese (*p* < 0.05). These findings are consistent with previous studies reporting reductions in short- and medium-chain saturated fatty acids following olive by-product supplementation [15,16,17]. In the present study, this response was observed for MCFA in ripened Pecorino cheese, but not for SCFA, and was not detected in Ricotta cheese.

Among the long-chain fatty acids, PDOC supplementation increased linoleic acid (C18:2 n-6) in Pecorino cheese (*p* < 0.05), while reducing rumenic acid (C18:2 c9,t11; RA/CLA) in both Pecorino (*p* < 0.05) and Ricotta (*p* < 0.01). In contrast, trans-vaccenic acid (TVA), oleic acid, and α-linolenic acid (ALA) were not affected by dietary treatment in either dairy product (*p* > 0.05). The absence of a significant effect on TVA, despite the reduction in CLA, suggests that the response was not consistent across individual C18:1 and C18:2 fatty acids.

At the fatty acid group level, neither SFA nor total PUFA was affected by dietary treatment in Pecorino cheese. In Ricotta, however, total PUFA decreased in the EXP group (*p* < 0.05), although neither n-3 nor n-6 PUFA differed between treatments. The n-3/n-6 ratio was also lower in EXP Pecorino cheese (*p* < 0.05), reflecting the combined changes in individual fatty acids rather than a significant alteration in total n-3 or n-6 PUFA.

In previous studies, olive cake supplementation generally increased oleic acid and total monounsaturated fatty acids (MUFA), while reducing saturated fatty acids (SFA) and the atherogenicity index in ewe [17] and cow [15] milk. However, these responses have not been consistent across studies. Calabrese et al. [59] reported that supplementation of dairy cow diets with the same PDOC reduced total MUFA content in Provola cheese, without affecting total fatty acid or SFA contents, despite significant changes in individual fatty acids and lipid health indices.

Several factors may explain the lack of dietary effects on the cheese fatty acid profile observed in the present study. First, the forage-to-concentrate ratio (75:25) was considerably higher than that adopted in previous studies, in which concentrates accounted for a greater proportion of total dry matter intake [15]. Consequently, the inclusion of the same proportion of olive cake in the concentrate resulted in a lower contribution of olive cake-derived fatty acids to total dietary fatty acid intake. Second, the ewes were managed under continuous grazing, whereas previous studies involved either partial grazing [60,61] or total mixed ration feeding [17]. Because fresh pasture is naturally rich in unsaturated fatty acids, its substantial contribution to total dietary fatty acid intake may have reduced the potential to detect additional effects of olive cake supplementation. Third, the Pecorino cheese was ripened for 6 months, considerably longer than the fresh or 60-day cheeses evaluated in earlier studies [15,17,59,61]. The extended ripening period may have promoted lipolytic processes that altered the fatty acid profile independently of dietary treatment. Collectively, differences in feeding system, forage-to-concentrate ratio, and ripening time may help explain the absence of significant dietary effects on the fatty acid profile of Pecorino and Ricotta cheeses, as well as the variability reported across studies. Interestingly, some of the ewes involved in the present feeding trial were subsequently slaughtered, and their meat was used in cured-meat processing trials. Analysis of the fatty acid composition of meat fat revealed a significant enrichment in unsaturated fatty acids, particularly oleic acid, in meat and salami from ewes fed PDOC [7]. This finding suggests that the effects of PDOC supplementation on fatty acid deposition may be tissue-specific and may be more readily detectable in adipose tissues than in milk fat or dairy products under the feeding and processing conditions adopted in the present study.

### 3.5. Evolution of Microbial Populations During Cheese Making Assessed by Culture-Dependent Approach

Table 7 reports the results of plate count analyses performed throughout the cheese-making process, from the wooden vat biofilm to cheese after six months of ripening. The wooden vat biofilm exhibited high microbial loads, with total mesophilic microorganisms (TMM) and lactic acid bacteria (LAB) with coccus morphology, both mesophilic and thermophilic, exceeding 10^6^ CFU/cm^2^. These findings confirm that wooden surfaces act as reservoirs of autochthonous microbiota, as widely documented in traditional Sicilian PDO cheese production systems [21,64]. Significant differences between CTR and EXP dietary treatments were observed in raw milk. Specifically, TMM and the main mesophilic and thermophilic LAB groups were approximately one logarithmic cycle higher in milk from the EXP group, suggesting that animal diet can influence the microbial composition of milk at milking. This is consistent with previous studies highlighting the role of feeding and management practices in shaping milk microbiota [65,66]. However, following contact with the wooden vat, no significant differences were detected between CTR and EXP productions. This apparent homogenization can be attributed to the dominant and stable microbiota harboured by the wooden surfaces, which standardize the microbial profile of milk regardless of its initial composition. Similar effects have been reported in traditional cheese-making, where resident biofilms act as a natural inoculum [67,68].

As cheese-making progressed, the microbial composition of both fresh cheese and cheese after six months of ripening remained comparable between the two dietary treatments. This convergence is likely driven by the strong selective pressure exerted by LAB during fermentation and ripening, which rapidly dominate the microbial ecosystem through acidification and competition for nutrients [69,70]. During ripening, additional factors including progressive pH reduction, decreased water activity, and limited oxygen availability further contribute to the establishment of a stable and homogeneous microbial community, as widely reported for traditional sheep cheeses [71,72]. Notably, no pathogenic microorganisms relevant to dairy products, such as *Listeria monocytogenes*, *Salmonella* spp., or coagulase-positive staphylococci (CPS), were detected in any of the samples analysed throughout the production process. Therefore, these data were not included. Overall, these findings indicate that the dietary treatment did not compromise the microbiological safety of the final cheese, in agreement with previous studies on traditional cheeses produced using silages derived from prickly pear (*Opuntia* spp.) by-products [73].

### 3.6. Dynamics of Microbial Populations Investigated Through Illumina Technology

**Figure 1:**
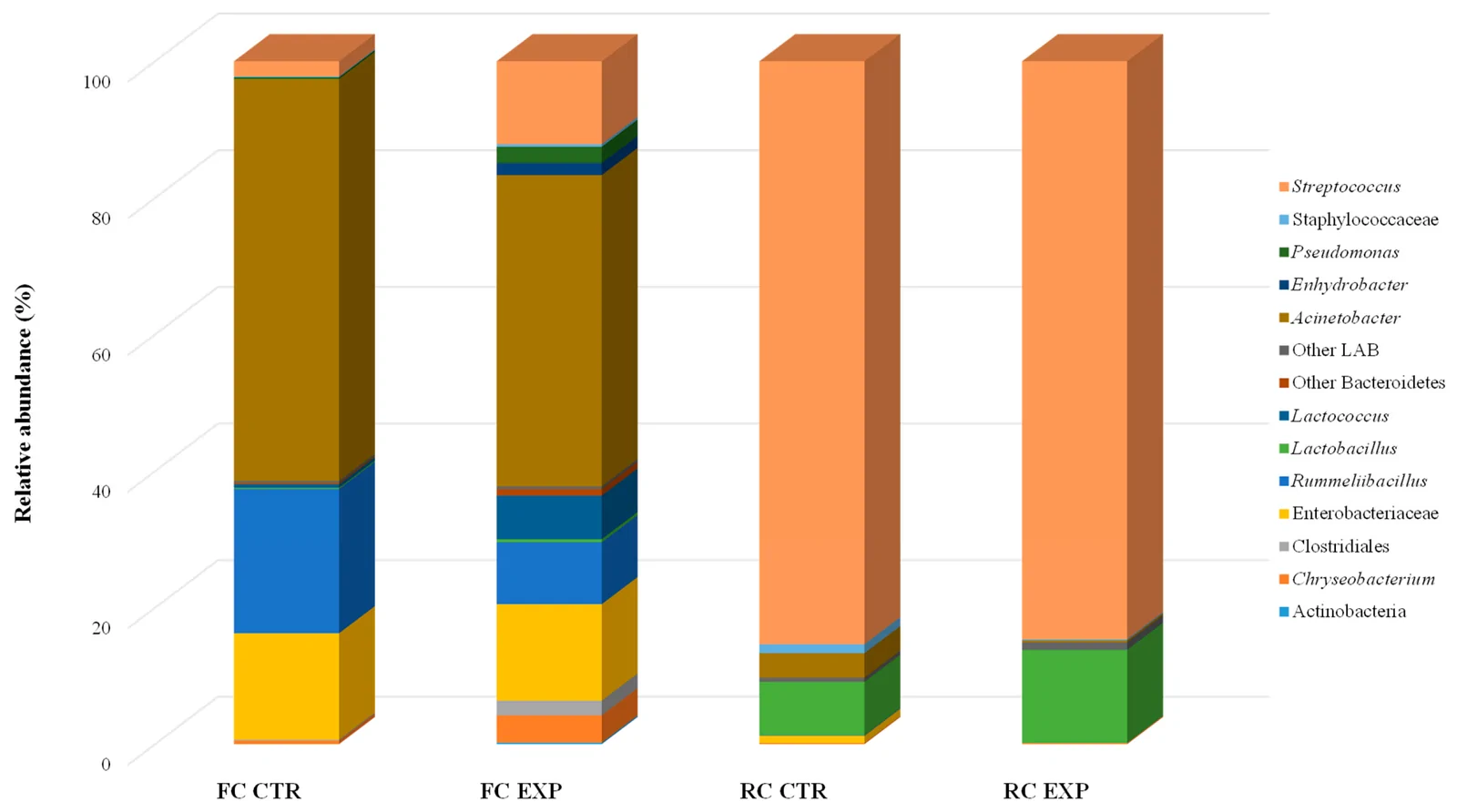
Relative abundances (%) of the bacterial operational taxonomic units (OTUs) identified using Illumina MiSeq sequencing. Abbreviations: FC CTR, fresh cheese control diet; FC EXP, fresh cheese experimental diet; RC CTR, ripened cheese control diet; RC EXP, ripened cheese experimental diet.

Analysis of the bacterial communities by high-throughput sequencing of the 16S rRNA gene (Figure 1) showed a complex microbial profile in fresh cheeses from both the CTR and EXP groups. After six months of ripening, the bacterial composition had undergone substantial changes. Overall, two phyla, two orders, two families, and eight genera were identified. Figure 1 shows only the taxa exhibiting a relative abundance (RA) greater than 0.1%. Fresh cheeses from both production groups were characterized by a marked predominance of *Gammaproteobacteria*, with *Acinetobacter* representing the most abundant genus, accounting for 58% and 45% of the total bacterial community in CTR and EXP samples, respectively. *Acinetobacter* is a well-known psychrotrophic genus commonly associated with food spoilage during refrigerated storage and is frequently detected in both food products and processing environments. Its high prevalence in fresh dairy products is therefore consistent with previous reports [73,74,75,76,77,78]. Enterobacteriaceae represented the second most abundant bacterial group in fresh cheeses, with relative abundances of 15.58% in CTR samples and 14.11% in EXP samples. However, this bacterial family was no longer detected in cheeses after six months of ripening, likely as a consequence of adverse environmental conditions such as low pH, increased salinity, and reduced water activity [79]. After six months of ripening, NGS analysis indicated that the bacterial communities of both CTR and EXP cheeses were largely dominated by LAB, particularly members of the genera *Streptococcus* and *Lactobacillus*. *Streptococcus* reached 85.36% RA in CTR cheeses and 84.61% RA in EXP cheeses. Although *Streptococcus* is a typical component of LAB starter cultures [80], the high relative abundance detected by sequencing may partly reflect the persistence of DNA from dead or viable-but-non-culturable cells, a known limitation of DNA-based approaches previously reported in Sicilian cheeses [21]. Lower relative abundances of *Lactobacillus* were observed in ripened cheeses from both productions. However, their presence is expected, as these bacteria are commonly encountered in cheeses manufactured from raw ewe’s milk [81].

### 3.7. Sensory Profile of Ripened Pecorino Siciliano PDO Cheese

**Figure 2:**
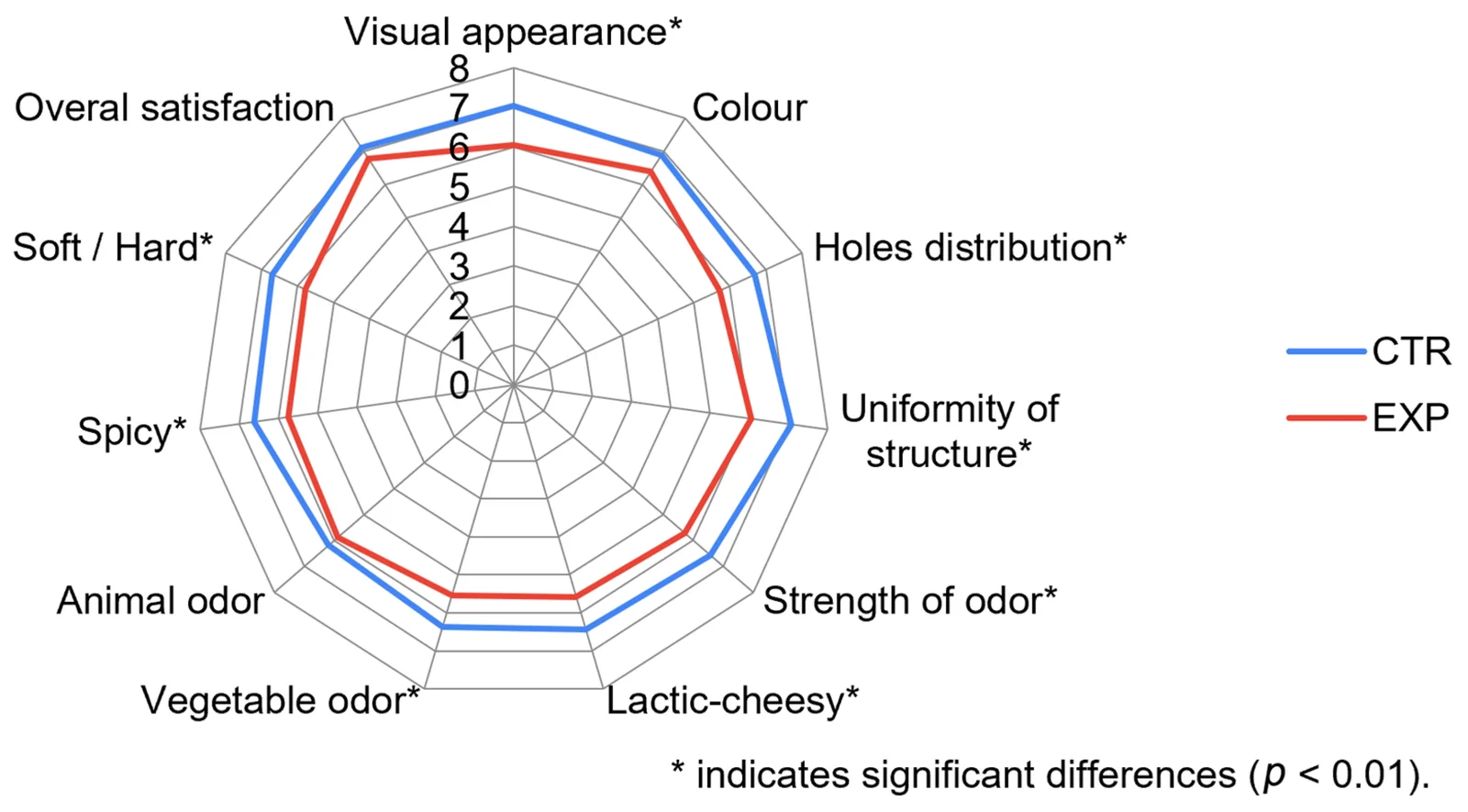
Spider plot of the descriptive sensory analysis of PDO Pecorino cheeses. Abbreviations: CTR = control ewes group fed on concentrate without olive cake; EXP = experimental ewes group fed on concentrate with supplementation of olive cake.

The sensory profiles of ripened cheeses are presented in Figure 2. Sensory evaluation is an essential step in the assessment of dairy products prior to commercialization, as alternative feeding strategies and production practices may influence consumer perception and product acceptability [82]. The sensory properties of cheese are affected by several factors, including animal diet, farm management practices [83], and the microbiological quality of milk used for cheesemaking [84].

With the exception of hole distribution and vegetable odor, cheeses produced from the milk of ewes receiving the PDOC-supplemented diet generally received lower scores for visual appearance, structural uniformity, odor intensity, lactic and cheesy odor, spicy note, and texture than those from the CTR group. Despite these differences in individual sensory attributes, the two cheese types showed comparable overall acceptability, indicating that PDOC supplementation altered specific sensory characteristics without negatively affecting consumer appreciation. In contrast, Chiofalo et al. [15] reported greater consumer acceptance of cheese produced from the milk of dairy cows supplemented with PDOC at 15% of dietary dry matter. This discrepancy may be attributed to differences in animal species, feeding system, cheesemaking technology, and ripening conditions, as Chiofalo et al. [15] evaluated a semi-ripened cow milk cheese, whereas the present study focused on a 6-month-ripened ewe milk Pecorino cheese.

**Table 2:** Chemical composition of diets and ingredients used to formulate diets (means ± SD) adapted from Maniaci et al. [7].

Items	Concentrate		Pasture	PDOC
	CTR	EXP		
Dry Matter, %	88.72 ± 0.32	89.20 ± 0.24	21.42 ± 8.00	93.91 ± 0.22
Crude Protein, % of DM	19.06 ± 0.48	20.97 ± 0.28	14.92 ± 3.54	11.50 ± 0.34
Ether extract, % of DM	2.75 ± 0.08	5.68 ± 0.39	2.28 ± 0.32	21.70 ± 0.55
aNDFom, % of DM	32.19 ± 2.58	36.48 ± 1.14	46.06 ± 9.47	62.27 ± 1.10
ADFom, % of DM	15.23 ± 1.74	16.70 ± 1.06	28.90 ± 6.02	48.78 ± 0.54
ADL, % of DM	2.04 ± 0.08	3.88 ± 0.05	4.77 ± 1.36	22.08 ± 1.70
Ash, % of DM	5.76 ± 0.43	4.97 ± 0.18	2.98 ± 0.57	3.40 ± 0.65
NFC	40.25 ± 2.99	31.89 ± 1.38	24.43 ± 5.95	1.14 ± 0.12
NE_L_ (kcal/kg DM) *	1872 ± 27.3	1800 ± 23.6	-	-
NE_L_ (UFL/kg DM) *	1.06 ± 0.03	1.02 ± 0.02	-	-
C16:0	18.65 ± 0.92	17.78 ± 0.84	18.37 ± 1.45	19.14 ± 1.07
C18:0	1.58 ± 0.18	2.23 ± 0.27	2.23 ± 0.53	2.91 ± 0.25
C18:1 n-9 OA	19.27 ± 1.05	35.55 ± 1.27	10.63 ± 4.50	55.56 ± 1.29
C18:2 n-6 LA	51.49 ± 3.17	35.67 ± 2.41	24.23 ± 8.36	12.36 ± 1.23
C18:3 n-3 ALA	5.21 ± 0.43	3.13 ± 0.27	35.38 ± 10.61	0.76 ± 0.09
SFA	20.98 ± 0.83	20.84 ± 0.77	23.92 ± 2.38	22.94 ± 1.13
MUFA	21.54 ± 1.12	38.36 ± 1.57	12.73 ± 5.27	59.87 ± 1.31
PUFA	57.48 ± 2.26	40.80 ± 2.17	63.35 ± 7.52	17.20 ± 1.49
Polyphenols mg GAE/g DM	3.79 ± 0.47	5.60 ± 0.06	14.34 ± 3.21	7.52 ± 0.44

* NE_L_ = net energy for lactation [85]; UFL = net energy content of 1 kg of standard barley for milk production, which is equivalent to 1700 kcal; CTR = control diet; EXP = experimental diet; PDOC = partially destoned olive cake; NDFom = NDF organic matter. ADFom = ADF organic matter; NFC = 100 − (CP + ether extract + ash + NDFom). OA = oleic acid; LA = linoleic acid; ALA = α-linolenic acid; SFA: saturated fatty acids; MUFA: monounsaturated fatty acids; PUFA: polyunsaturated fatty acids; GAE = gallic acid equivalent.

**Table 3:** Physicochemical parameters of bulk milk used for cheesemaking.

Items	Bulk Milk		SEM	*p*-Value
	CTR	EXP		
Fat (%)	6.01	6.15	0.289	0.063
Protein (%)	5.40	5.38	0.041	0.629
Casein (%)	4.11	4.09	0.034	0.571
Non-protein N (%)	0.038	0.029	0.010	0.551
Whey protein (%)	1.08	1.38	0.139	0.176
Lactose (%)	4.66	4.66	0.035	0.999
Urea (mg/dL)	40.51	39.64	0.709	0.178
SCC (log10)	5.92	6.19	0.087	0.039
TBC (log10 cfu/mL)	5.53	5.80	0.167	0.069
pH	6.70	6.73	0.017	0.064
r (min)	20.56	21.10	1.019	0.401
a30 (mm)	42.35	45.58	2.229	0.013
K20 (min)	2.25	1.71	0.252	0.031

CTR = control diet; EXP = experimental diet; SEM = standard error of the means; SCC = somatic cell count; TBC = total bacterial count; r (min) = rennet coagulation time; a30 (mm) = curd firmness at 30 min; K20 (min) = curd firming time.

**Table 4:** Physical properties and chemical composition of Ricotta and ripened Pecorino Siciliano PDO cheeses.

Items	PDO Cheeses		SEM	*p*-Value	Ricotta		SEM	*p*-Value
	CTR	EXP			CTR	EXP		
DM (%)	69.44	70.19	1.400	0.414	30.78	31.91	1.51	0.108
CP (%DM)	43.24	43.45	0.867	0.868	31.49	30.50	1.66	0.657
EE (%DM)	45.06	44.91	0.72	0.798	56.83	57.74	2.694	0.803
Ash (%DM)	9.11	9.44	0.296	0.328	4.86	4.86	0.243	0.980
POV (mEq O_2_/kg fat)	2.26	2.96	0.445	0.316	2.05	1.94	0.169	0.673
TPC (mg GAE/g DM)	7.46	7.34	0.175	0.649	3.26	2.61	0.079	0.002
TBARS (mg/kg DM)	0.20	0.24	0.048	0.568				
Titratable acidity	0.43	0.42	0.028	0.716				
L*, lightness	74.28	70.46	2.503	0.331				
a*, redness	−3.45	−3.65	0.261	0.437				
b*, yellowness	11.02	11.38	0.283	0.429				
Hardness (kg/cm^2^)	0.80	0.92	0.115	0.285				

CTR = control diet; EXP = experimental diet; SEM = standard error of means; DM = dry matter; CP = crude protein; EE = ether extract; TBARS = thiobarbituric acid reactive substance; POV = peroxide value; TPC = total phenolic content.

**Table 5:** Total fatty acids (% DM) and short- and medium-chain fatty acid profiles (g/100 g FA) of Ricotta and ripened Pecorino Siciliano PDO cheeses.

Fatty Acid (FA)	Pecorino Cheese		SEM	*p*-Value	Ricotta Cheese		SEM	*p*-Value
	CTR	EXP			CTR	EXP		
Total FA, % DM	45.26	43.78	1.581	0.537	53.06	52.13	2.690	0.763
C4:0	2.12	2.37	0.315	0.562	2.54	2.40	0.128	0.475
C6:0	2.79	2.56	0.130	0.259	2.49	2.51	0.989	0.868
C8:0	2.86	2.63	0.172	0.312	2.49	2.47	0.152	0.778
C10:0	8.12	7.61	0.492	0.278	7.01	7.17	0.445	0.234
C10:1	0.37	0.34	0.019	0.027	0.32	0.32	0.020	0.940
C12:0	4.40	4.21	0.244	0.143	4.10	4.20	0.265	0.234
C12:1	0.18	0.17	0.011	0.004	0.05	0.04	0.003	0.137
C14:0 iso	0.14	0.13	0.004	0.232	0.30	0.30	0.013	0.596
C14:0	10.31	10.43	0.181	0.669	10.88	11.08	0.170	0.453
C14:1	1.01	0.97	0.032	0.033	0.66	0.65	0.018	0.233
C15:0 iso	0.32	0.32	0.013	0.990	0.31	0.31	0.010	0.667
C15:0	1.33	1.32	0.026	0.693	1.33	1.33	0.023	0.775
C15:1	0.16	0.17	0.015	0.674	0.26	0.26	0.400	0.865
C16:0	22.29	23.32	0.834	0.390	24.46	24.55	0.736	0.707
C16:1	1.64	0.99	0.143	0.023	1.72	1.66	0.032	0.003
C17:0	0.80	0.82	0.030	0.637	0.33	0.30	0.019	0.162
C17:1	0.21	0.21	0.011	0.772	0.19	0.17	0.019	0.369
C17:0 anteiso	0.34	0.33	0.022	0.710	1.26	1.19	0.069	0.269

CTR = control diet; EXP = experimental diet; SEM = standard error of means; FA = fatty acid.

**Table 6:** Long-chain fatty acid profile (g/100 g FA) and nutritional lipid indices of Ricotta and ripened Pecorino Siciliano PDO cheeses.

Fatty Acids	Pecorino Cheese		SEM	*p*-Value	Ricotta Cheese		SEM	*p*-Value
	CTR	EXP			CTR	EXP		
C18:0	10.07	10.49	0.064	0.501	9.97	10.15	0.390	0.241
C18:1 t11, TVA	2.92	2.55	0.310	0.155	2.86	3.27	0.437	0.260
C18:1 c9 OA	16.18	17.10	0.707	0.106	19.69	19.07	0.602	0.147
C18:2 n 6, LA	1.91	2.06	0.117	0.044	1.92	1.92	0.123	0.968
C18:2 c9 t11 RA, CLA	1.21	1.00	0.123	0.047	0.93	0.82	0.108	0.008
C18:3 n-6, GLA	0.06	0.06	0.004	0.944	0.24	0.25	0.025	0.296
C18:3 n-3, ALA	1.14	1.09	0.065	0.514	0.92	0.90	0.068	0.289
C19:0	0.40	0.36	0.031	0.279	0.15	0.14	0.019	0.220
C20:0	0.27	0.29	0.028	0.269	0.10	0.09	0.010	0.050
C20:4 n-6, AA	0.10	0.10	0.008	0.595	0.09	0.09	0.010	0.796
C20:5 n-3, EPA	0.13	0.13	0.003	0.380	0.09	0.09	0.014	0.849
C22:0	0.15	0.15	0.010	0.436	0.13	0.13	0.012	0.231
SFA	66.81	67.44	0.288	0.108	67.85	68.31	0.216	0.148
MUFA	27.49	27.14	0.130	0.050	27.40	27.09	0.221	0.257
PUFA	5.70	5.42	0.217	0.258	4.75	4.60	0.114	0.027
n-6 PUFA	2.26	2.41	0.046	0.393	2.37	2.38	0.134	0.906
n-3 PUFA	1.27	1.21	0.064	0.503	1.02	0.99	0.074	0.321
n-3/n-6	0.57	0.52	0.202	0.043	0.44	0.42	0.051	0.224
SCFA	7.77	7.56	0.396	0.719	7.51	7.37	0.295	0.696
MCFA	26.34	25.66	0.766	0.024	22.67	23.11	0.839	0.234
LCFA	65.89	66.78	0.922	0.285	67.25	66.96	1.036	0.392
Thrombogenic index	2.22	2.34	0.082	0.281	2.47	2.53	0.063	0.182
Health promoting Index	0.49	0.47	0.012	0.357	0.45	0.43	0.006	0.206

CTR = control diet; EXP = experimental diet; SEM = standard error of means; TVA = trans vaccenic acid; RA = rumenic acid; LA = linoleic acid; CLA = conjugated linoleic acid; GLA = γ-linolenic acid; ALA = α-linolenic acid; AA = arachidonic acid; EPA = eicosapentaenoic acid; SCFA = short chain fatty acids; MCFA = medium chain fatty acids; LCFA = long-chain fatty acids; Thrombogenic index = (C14:0 + C16:0 + C18:0)/(0.5 × MUFA + 0.5 × n-6 PUFA + 3 × n-3 PUFA + n-3/n-6) [86]. Health-promoting index = (n-3 PUFA + n-6 PUFA + MUFA)/(C12:0 + 4 × C14:0 + C16:0) [87].

**Table 7:** Microbial evolution during cheese production.

Samples	Growth Media						
	SMA 30 °C	MRS 30 °C	M17 30 °C	MRS 44 °C	M17 44 °C	BP	*E. coli*
Wooden vat surface	6.35 ± 0.71	4.09 ± 0.16	6.16 ± 0.14	4.65 ± 0.19	6.22 ± 0.17	<1	1.57 ± 0.11
Bulk Milk (BM)							
CTR	4.98 ± 0.04 ^B^	3.40 ± 0.14 ^B^	4.49 ± 0.23 ^B^	3.40 ± 0.14 ^B^	4.05 ± 0.10 ^B^	1.98 ± 0.22	1.38 ± 0.25
EXP	5.39 ± 0.16 ^A^	4.53 ± 0.18 ^A^	5.21 ± 0.14 ^A^	4.72 ± 0.25 ^A^	5.13 ± 0.18 ^A^	2.02 ± 0.13	1.30 ± 0.28
*p* value	0.045	0.007	0.037	0.009	0.006	0.847	0.794
Milk after contact (MAC)							
CTR	6.08 ± 0.25	4.35 ± 0.35	5.97 ± 0.10	4.27 ± 0.23	5.57 ± 0.40	2.06 ± 0.16	1.64 ± 0.21
EXP	6.16 ± 0.17	4.93 ± 0.11	6.03 ± 0.18	4.36 ± 0.18	5.70 ± 0.28	2.30 ± 0.18	2.02 ± 0.14
*p* value	0.746	0.124	0.722	0.707	0.745	0.274	0.137
Curd (C)							
CTR	6.51 ± 0.21	6.18 ± 0.25	6.31 ± 0.29	5.39 ± 0.30	5.25 ± 0.21	3.01 ± 0.14	3.00 ± 0.15
EXP	6.75 ± 0.14	6.61 ± 0.52	6.60 ± 0.42	5.69 ± 0.30	5.70 ± 0.14	3.10 ± 0.10	3.29 ± 0.39
*p* value	0.292	0.391	0.501	0.413	0.097	0.533	0.420
Fresh cheese (FC)							
CTR	7.32 ± 0.31	7.21 ± 0.14	7.29 ± 0.29	7.24 ± 0.11	7.35 ± 0.10	3.20 ± 0.17	3.24 ± 0.21
EXP	7.54 ± 0.23	7.65 ± 0.42	7.28 ± 0.18	7.35 ± 0.18	7.77 ± 0.12	3.43 ± 0.15	3.49 ± 0.16
*p* value	0.500	0.275	0.971	0.535	0.137	0.267	0.294
Ripened cheese (RC)							
CTR	7.95 ± 0.16	7.36 ± 0.24	7.80 ± 0.14	7.63 ± 0.19	7.94 ± 0.21	<2	<2
EXP	7.99 ± 0.15	7.41 ± 0.12	7.89 ± 0.19	7.74 ± 0.26	8.01 ± 0.18	<2	<2
*p* value	0.822	0.819	0.644	0.678	0.757	n.e.	n.e.

Loads are reported as log CFU/cm^2^ for vat surface, log CFU/mL for milk samples, and log CFU/g for curd and cheese. Results indicate the mean values ± standard deviation (S.D.) of twelve plate counts (carried out in duplicate for six independent productions). Data within a row followed by different letters are significantly different according to Tukey’s test. Abbreviations: SMA, skim milk agar for detection of total mesophilic microorganisms; MRS 30 °C, de Man–Rogosa–Sharpe agar for mesophilic rod LAB; M17 30 °C, medium 17 agar incubated at 30 °C for mesophilic coccus LAB; MRS 44 °C, de Man–Rogosa–Sharpe agar medium for thermophilic rod LAB; M17 44 °C, medium 17 agar incubated at 44 °C for thermophilic coccus LAB; BP, Baird-Parker agar for detection of coagulase-positive staphylococci; *E. coli*, *Escherichia coli*; CTR, control diet; EXP, experimental diet; n.e., not evaluated.

## 4. Conclusions

The inclusion of stabilized, partially destoned olive cake (17%, as-fed basis) in the concentrate of grazing dairy ewes did not affect bulk milk composition and improved milk coagulation properties. In Pecorino Siciliano PDO cheese, PDOC supplementation reduced the proportions of some medium-chain unsaturated fatty acids and the omega-3/omega-6 ratio, while overall sensory acceptability of Pecorino Siciliano PDO cheese was not affected. Although the microbial communities of raw milk differed between dietary treatments, those of ripened Pecorino Siciliano PDO cheese converged during cheesemaking and ripening, highlighting the predominant influence of cheesemaking and ripening processes over dietary treatment on the final cheese microbiota.

The absence of significant dietary effects on the overall fatty acid profile and oleic acid content of cheese contrasts with findings from previous studies and may be related to differences in forage-to-concentrate ratio, grazing management, olive cake processing, and the extended ripening period adopted in the present trial. A limitation of the present study is that only one inclusion level of PDOC was evaluated, preventing assessment of possible dose-dependent effects. Nevertheless, the 17% inclusion level was selected based on previous evidence in dairy sheep, which indicated its suitability without detrimental effects on animal performance and milk production. Further studies evaluating different inclusion levels would therefore be useful to identify the optimal dietary level of stabilized, partially destoned olive cake and to better characterize potential dose-dependent effects on milk and cheese quality.

Overall, stabilized, partially destoned olive cake represents a promising sustainable feed ingredient for grazing dairy ewes, as its inclusion at 17% in the concentrate maintained the technological and microbiological quality of Pecorino Siciliano PDO and the compositional and microbiological quality of Ricotta, while overall sensory acceptability of Pecorino Siciliano PDO cheese was not affected, although some changes in the nutritional characteristics of cheese were observed.

## Figures and Tables

**Table 1:** Ingredients used to formulate concentrates offered to ewes.

Ingredients (%)	CTR	EXP
Partially destoned olive cake (PDOC)	-	17
Soybean meal (48%)	13	15
Corn grains	5	17
Beet pulp	50	30
Wheat flour	-	21
Wheat bran	32	-

## Data Availability

The datasets used and/or analysed during the current study are available from the corresponding author upon reasonable request.
